# 7T MRI in epilepsy patients with previously normal clinical MRI exams compared against healthy controls

**DOI:** 10.1371/journal.pone.0213642

**Published:** 2019-03-19

**Authors:** Rebecca Emily Feldman, Bradley Neil Delman, Puneet Singh Pawha, Hadrien Dyvorne, John Watson Rutland, Jiyeoun Yoo, Madeline Cara Fields, Lara Vanessa Marcuse, Priti Balchandani

**Affiliations:** 1 Translational and Molecular Imaging Institute, Icahn School of Medicine at Mount Sinai, New York, New York, United States of America; 2 Radiology, Icahn School of Medicine at Mount Sinai, New York, New York, United States of America; 3 Department of Neurology, Mount Sinai Hospital, New York, New York, United States of America; McGill University, CANADA

## Abstract

**Objective:**

To compare by 7 Tesla (7T) magnetic resonance imaging (MRI) in patients with focal epilepsy who have non-lesional clinical MRI scans with healthy controls.

**Methods:**

37 patients with focal epilepsy, based on clinical and electroencephalogram (EEG) data, with non-lesional MRIs at clinical field strengths and 21 healthy controls were recruited for the 7T imaging study. The MRI protocol consisted of high resolution T_1_-weighted, T_2_-weighted and susceptibility weighted imaging sequences of the entire cortex. The images were read by two neuroradiologists, who were initially blind to clinical data, and then reviewed a second time with knowledge of the seizure onset zone.

**Results:**

A total of 25 patients had findings with epileptogenic potential. In five patients these were definitely related to their epilepsy, confirmed through surgical intervention, in three they co-localized to the suspected seizure onset zone and likely caused the seizures. In seven patients the imaging findings co-localized to the suspected seizure onset zone but were not the definitive cause, and ten had cortical lesions with epileptogenic potential that did not localize to the suspected seizure onset zone. There were multiple other findings of uncertain significance found in both epilepsy patients and healthy controls. The susceptibility weighted imaging sequence was instrumental in guiding more targeted inspection of the other structural images and aiding in the identification of cortical lesions.

**Significance:**

Information revealed by the improved resolution and enhanced contrast provided by 7T imaging is valuable in noninvasive identification of lesions in epilepsy patients who are non-lesional at clinical field strengths.

## Introduction

Approximately 20–40% of individuals with epilepsy do not respond to anti-seizure drug therapy [1–3]. For these patients, surgery may offer the best chances for seizure freedom [4]. Magnetic resonance imaging (MRI) exams are a key element of the pre-surgical epilepsy work-up. Clear identification of lesions on an MRI exam, when concordant with electrophysiology and clinical measures, results in more accurate surgical interventions and better outcomes [5–8].

However, 20%-30% of patients with focal epilepsy are “MRI-negative,” meaning that they do not have an identifiable lesion on MRI [1, 9, 10]. MRI-negative patients are less likely to be considered candidates for surgery than lesional patients and when operated upon have inferior surgical outcomes overall [11, 12]. MRI-negative individuals who do undergo successful surgery frequently have distinct epileptogenic lesions identified post-surgery via histopathological investigations or retrospective examination of the images [13, 14]. Since reduced surgical success for MRI-negative individuals is often attributable to inaccurate or incomplete resection of epileptogenic foci [13–17], pre-surgical identification of abnormalities on MRI may be an important contributor to positive surgical treatment outcomes.

Increased field strength provides an advantage in imaging for epilepsy treatment planning [13, 18–22]. However, even at 3 Tesla (3T), many individuals with focal epilepsy remain MRI-negative [20]. Ultra-high field MRI scanners, such as units operating at 7 Tesla (7T), offer improved signal to noise ratio or higher image resolution, which enhances the conspicuity of epileptogenic lesions and provide more accurate delineation of lesion boundaries. 7T neurological imaging protocols are noninvasive and well tolerated by patients [23]. The U.S. Food and Drug Administration has designated MRI scanners at 8 Tesla and below as non-significant risk [23] and 7T models approved for clinical use are operational.

7T has been useful in identifying hippocampal architecture and sclerosis [24–27], cortical dysplasia [19], and vascular malformations [28]. It has enabled improved visualization of the amygdalohippocampal border [29] as well as polymicrogyria [30, 31]. Recent work has shown the value of 7T in resolving potentially epileptogenic abnormalities in 21 epilepsy patients with non-contributory conventional strength MRI exams [32, 33].

The improved resolution or contrast of the 7T is valuable not only in elucidating vascular malformations, hippocampal, and cortical lesions, but it may also be used to identify subtle structural abnormalities previously beyond the threshold of detectability and potentially related to epilepsy. However, in order to differentiate findings potentially related to epilepsy from non-pathological features, a comparison to benign abnormalities detected in healthy controls at 7T is required. We report results for the first controlled study designed to assess the ability of 7T imaging to reveal subtle abnormalities in 37 patients with focal epilepsy who have non-lesional diagnostic MRI scans performed at conventional clinical field strengths. We evaluate the specific contributions of each pulse sequence at 7T and compare the abnormalities detected in non-lesional epilepsy patients to those observed in healthy controls.

## Methods

### Experiment

Institutional Review Board (IRB) approval for human research was obtained for this experiment from the Program for Protection of Human Subjects at the Icahn School of Medicine at Mount Sinai. Written informed consent was obtained from each participant. Each participant was asked to read an IRB approved consent document explaining the experiment, the participant’s role in the experiment, and the participant’s rights before, during, and after the experiment. Next, the participant’s role in the experiment was summarized by a researcher and the participant was given an opportunity to have any questions answered. Finally, the participant was asked to provide written consent by signing the consent documents. These documents are kept secured at Mount Sinai.

Between July 2014 and October 2016, patients with epilepsy were recruited for the study by three epileptologists. Patients eligible for the study were patients with definite focal epilepsy and a non-lesional clinical MRI. Definite epilepsy was based on the clinical history and the electroencephalogram (EEG) (Table 1). A normal EEG was not an exclusion criteria. Exclusion criteria included generalized epilepsy, a lesional clinical MRI, traumatic brain injury or another central nervous system disease like Alzheimer's disease. If a patient did not definitively have focal epilepsy, they were not included in the study.

**Table 1:** Summary of clinical details for epilepsy patients and EEG results.

ID	Seizure Types ILAE Classification	EEG Results	Age at Diagnosis
1	**FAS,** cognitive **FIAS**, frequent	Frequent L FT slowing Occasional L FT spikes	21
2	**FIAS,** frequent, hyperkinetic nocturnal **FTBTCS**, frequent	Background normal Multiple hyperkinetic seizures Non-lateralizable	13
3	**FIA,** rare **FTBTCS,** rare	B/l FT slowing B/l hemispheric sharp waves L > R	30
4	**FIAS,** rare **FTBTCS,** rare	L hemispheric spikes, maximal anteriorly	55
5	**FAS,** motor, frequent L arm proximal jerking, rare R arm jerking **FTBTCS,** rare	Frequent b/l R >> L hemispheric Para-sagittal sharp waves and spikes Seizure onset usually on R	5
6	**FAS,** cognitive **FIAS,** rare **FTBTCS,** lifetime 1	Rare R FT sharp waves	33
7	**FAS**, cognitive with spatial disorientation **FIAS,** frequent **FTBTCS,** occasional	Abundant R hemispheric sharp waves, spikes, and B(I)RDs Seizures not lateralizable on EEG **Intracranial EEG** multi-focal R hemispheric onset	19
8	**FAS,** sensory with visual phenomena **FIAS,** occasional	Rare L temporal slowing Rare L temporal sharp waves	28
9	**FAS**, cognitive **FIAS,** frequent **FTBTCS,** rare, lifetime 4	B/l FT spikes Seizure of L temporal onset	15
10	**FTBTCS,** rare, lifetime 5	Frequent R FT RDA Rare R frontal spikes	19
11	**FIAS,** cognitive	Fair organization GRDA+S L temporal sharp waves Diffuse sharp waves **Intracranial EEG** b/l multifocal epileptiform potentials No clear electrographic seizures	18
12	**FAS,** cognitive **FIAS,** non-motor with aphasia and garbled speech	GRDA+S Diffuse spikes L FT spikes Rare R FT spikes Seizure onsets differ on EEG Difficult to lateralize	13
13	**FIAS**, frequent **FTBTCS,** frequent	Occasional L temporal sharp waves Seizures with L temporal onset **Intracranial EEG** b/l hippocampus and amygdala sharp waves and spikes, 2 seizures of left sided onset	2
14	**FAS,** cognitive **FTBTCS** feels about to pass out, lifetime 2	Mild generalized slowing B/l temporal sharp waves	43
15	**FAS,** cognitive **FTBTCS,** nocturnal, rare	Normal	28
16	**FAS** motor, head moves to the R, occasional **FIAS** motor with confusion, frequent **FTBTCS** frequent	Frequent L central spikes FAS are EEG negative	14
17	**FAS,** sensory with visual hallucinations, frequent **FTBTCS** rare, lifetime history of 4	Occasional L parietal B(I)RDs Rare L parietal spikes Seizure not captured	8
18	**FAS**, cognitive, occasional **FIAS,** occasional, 6–8 per year	L anterior temporal sharp waves Intermittent bi-temporal slowing L temporal seizures	35
19	**FAS**, cognitive, frequent **FIAS**, frequent, 2–3 per week	L FT slowing L temporal quasi-periodic sharp waves L anterior temporal LRDA 3 seizures of L temporal origin	4
20	**FAS** motor, right facial twitching, frequent **FTBTCS,** rare	Frontal sharp waves and spikes L FC B(I)RDs and L RDA Frequent electroclinical seizures with R facial twitching L FC electrographic onset	29
21	**FIAS** behavior arrest, asystole, syncope, rare	R temporal sharp waves Rare L temporal spikes **Intracranial EEG** R multifocal spiking Seizures R LT	21
22	**FAS**, cognitive and sensory, cannot speak, drools, frequent, daily	Recent EEGs normal Past with multi-focal discharges	13
23	**FAS** cognitive, frequent, 0–2 per month	L LPD L temporal spikes L temporal lobe seizures captured on EEG	44
24	**FAS** cognitive, frequent **FIAS** frequent, 2–3 per month	R anterior temporal spikes R temporal seizures captured	6 months
25	**FIAS/FTBTCS,** motor onset, asynchronous bilateral movement, frequent, 2 or more per month	B/l slowing B/l temporal sharp waves Multiple seizures captured, non-lateralizable **Intracranial EEG** B/l independent mesial temporal onset	20
26	**FIAS** automatisms of right hand, frequent, 2–3 per month	B/l FT slowing B/l temporal sharp waves Unclear electrographic onset (one R and one L)	40
27	**FIAS,** cognitive with aphasia, occasional	L FT slowing; mild generalized slowing L temporal RDA Rare L temporal sharp waves	77
28	**FIAS,** emotional onset, behavioral arrest, or decreased responsiveness, prolonged, frequent **FTBTCS**, rare	15 year history of normal EEGs **After enrollment in study** Diffuse rhythmic theta Multiple seizures with decreased responsiveness 1 FBTCS not lateralizable electrographically	45
29	**FAS**, jerking of the right face, frequent	Normal	33
30	**FAS,** cognitive or sensory, frequent, daily **FIAS**, peri-oral automatisms, frequent, weekly **FTBTCS,** rare	Occasional R temporal slowing Frequent R temporal sharp waves Multiple seizures R sided onsets some temporal, some central	10
31	**FAS,** sensory **FIAS,** oral automatism, head to the R, R gaze deviation **FTBTCS,** rare	Background normal Seizure onset diffuse Better development on L	26
32	**FAS,** multiple onset sensory or cognitive, frequent **FIAS,** rare **FTBTCS,** rare	R anterior slowing GRDA+S 2 seizures of R temporal onset	20
33	**FAS,** sensory **FIAS,** confusion, frequent, 3–4 per month **FTBTCS,** rare	L posterior quadrant slowing L occipital onset seizures	17
34	**FIAS,** automatisms and behavioral arrest, frequent, 1 per month **FTBTCS,** rare	L FT slowing L RDA Sharp waves and spikes—L FT and b/l **Intracranial EEG** seizures L hippocampal-amygdala origin.	46
35	**FIAS,** cognitive, 7 clusters per year	Rare L temporal sharp waves 5 seizures: 4 with L FT onset; 1 non-lateralizable	19
36	**FIAS** sensory of right arm then shaking of the right arm. Eyes to the R then head turns to the left. Often nocturnal, frequent shaking of R arm, mouth automatisms **FTBTCS** rare	Abundant b/l central spikes more prominent on the L **Intracranial EEG** b/l spiking 12 seizures of onset within left parietal lesion Rapid spread	19
37	**FAS/FIAS,** cognitive onset to sensory, frequent, 3–4 per week	Rare R anterior temporal spikes	16

Abbreviations -> B(I)RDs: brief potentially ictal rhythmic discharges; B/l: bilateral; EPC: epilepsia partialis continua; FAS: focal aware seizure; FC: fronto-central; FIAS: focal impaired aware seizure; FT: fronto-temporal; FTBTCS: focal to bilateral tonic clonic seizure; GRDA: generalized rhythmic delta activity; ILAE: International League Against Epilepsy; L: left; LT: lateral temporal; LPD: lateralized periodic discharges; MT: medial temporal; R: right; RDA: rhythmic delta activity; +S plus spike/sharp-wave discharges

All patients had non-lesional diagnostic 1.5T or 3T MRI scans based on a clinical protocol that met or exceeded ‘Minimum Recommended Imaging’ in epilepsy [34]. We enrolled 37 epilepsy patients (20 male, 36 ±14 years) as well as 21 normal healthy controls (15 male, 34 ±10 years). All subjects were between 18–78 years old and had no contraindications to 7T MRI. Images were acquired on a 7T whole body MRI scanner (MAGNETOM, Siemens Erlangen), equipped with an SC72CD gradient coil, using a single channel transmitter and a 32-channel receive head coil (Nova Medical, Wilmington, MA). All 7T findings were conveyed to the referring epileptologists and any subsequent changes in surgical plan and patient outcome (Engel classification [35]) were recorded in cases that progressed to surgery.

The imaging protocol consisted of six sequences. Four were acquired at a coronal-oblique angle prescribed perpendicular to the angle of the long axis of the body of the hippocampus: 1) MP-RAGE, 2) MP2RAGE[36], 3) T_2_ TSE, and 4) FLAIR. Two sequences were acquired axially: 5) susceptibility weighted imaging (SWI) and 6) T_2_ TSE. Total scan time, including localizers, was approximately 55 minutes. In instances where degraded image quality due to factors such as head motion precluded accurate analysis of the cortex, sequence acquisition was repeated a maximum of one time. Details of the sequences are available in Table 2. This protocol was optimized for detection of epileptogenic foci by reference to literature [18, 19, 25, 26, 28, 29, 32] and in consultation with a CAQ neuroradiologist (BD) [37, 38].

**Table 2:** Full epilepsy 7T MRI protocol imaging parameters.

Sequence Name	MPRAGE	MP2RAGE	T2 TSE	FLAIR	T2 TSE	SWI
Orientation	Coronal Oblique	Coronal Oblique	Coronal Oblique	Coronal Oblique	Axial	Axial
Scan Time [min:sec]	7:05	7:26	6:14	4:32	6:50	7:30
Voxel Size [mm^3^]	0.7 x 0.7 x 0.7	0.8 x 0.8 x 0.8	0.4 x 0.4 x 2.0	0.7 x 0.7 x 3.0	0.4 x 0.4 x 2.0	0.2 x 0.2 x 1.5
Slice Number	224	224	60	40	40	80
FOV [mm^2^]	225 x 183	225 x 183	225 x 183	225 x 183	202 x 183	210 x 171
2D/3D	3D	3D	2D	2D	2D	2D
TR [ms]	3000	6000	6000	9000	6000	23
TE [ms]	2	5.1	69	123	69	14
TI (TI2) [ms]	1050	1050(3000)	N/A	2600	N/A	N/A
Flip Θ (Flip Θ 2) [⁰]	6	5(4)	150	180	150	12
Resolution	320 x 260	282 x 146	512 x 416	320 x 260	512 x 464	1024 x 832
BW [Hz/Pixel]	430	130	279	244	279	150
Concatenations	1	1	2	3	2	1
Slice Oversampling [%]	7.1	7.1	N/A	N/A	N/A	10
PAT	2	3	2	3	2	3
Reference Lines PE	24	32	31	29	31	32
Echo Spacing	4.6	10.4	9.84	11.2	9.84	N/A
Turbo Factor	N/A	N/A	11	11	11	N/A
Echo Trains per Slice	N/A	N/A	20	9	20	N/A

Abbreviations -> BW: bandwidth; FLAIR: fluid attenuated inversion recover; FOV: Field of View; MPRAGE: magnetization prepared rapid gradient echo; MP2RAGE: magnetization prepared 2 rapid gradient echos; N/A: not applicable; PAT: integrated parallel imaging technique; PE: Phase encode; SWI: susceptibility weighted imaging; T2 TSE: T2-weighted turbo spin echo; TE: echo time; TI: inversion time; TR: repetition time

Both the MPRAGE and the MP2RAGE sequences covered the entire brain with isotropic resolution. From the MP2RAGE sequence, a total of four image sets were produced: 1) Inversion time (TI) of 1050 ms, 2) TI of 3000 ms, 3) T_1_ maps, and 4) uniform-denoised images. The T_1_ maps and uniform-denoised images were calculated from both the TI = 1050 ms and TI = 3000 ms images. Finally, the SWI sequence produced four sets of images: 1) magnitude images and 2) phase images, which were used to create 3) SWIs and 4) minimum intensity projections (mIPs) through each set of five contiguous SWI slices.

### Analysis

Neuroradiologic assessment, including a visual assessment of structural symmetry, was performed by two blinded expert neuroradiologists (BD and PP) on all 7T images and exams, so that neither control or patient status nor seizure onset zone (in patients) was known at the time of the assessment. Disagreements were resolved by consensus. The neuroradiologic findings for epilepsy patients and healthy controls were compared using a Fisher exact test to differentiate features and artifacts typically found at 7T from unusual or pathological findings and the significance of the difference was reported. Images from the epilepsy patients were subsequently reassessed by a neuroradiologist (BD) after un-blinding to the clinically suspected seizure onset zone (sSOZ). 7T MRI findings were compared with the sSOZ suggested by the clinical and/or EEG data. The relationship of the patient’s epilepsy to their 7T results was divided into five categories: *definite* (7T lesion of epileptogenic potential that highly localizes to the sSOZ and is confirmed through surgical intervention); *likely* (7T lesion of epileptogenic potential which highly localizes to the sSOZ and is highly likely to cause the patient’s epilepsy); *possible* (7T lesion of epileptogenic potential which localizes to the patient’s sSOZ but is not the definitive cause); *uncertain* (7T lesion has epileptogenic potential but does not localize to the sSOZ and does not correspond to clinical and EEG data); *none* (no 7T lesion or lesion has no epileptogenic significance).

In epilepsy patients sensitivity was calculated based on the presence or absence of four radiological findings: (a) hippocampal and cortical abnormalities (identified on MPRAGE, MP2RAGE, T2TSE, or FLAIR), (b) vascular abnormalities (identified on SWI), (c) prominent perivascular spaces, and (d) other abnormalities. Specificity was calculated from the healthy control data based on the presence or absence of the same four categories of radiological findings.

## Results

**Fig 1:**
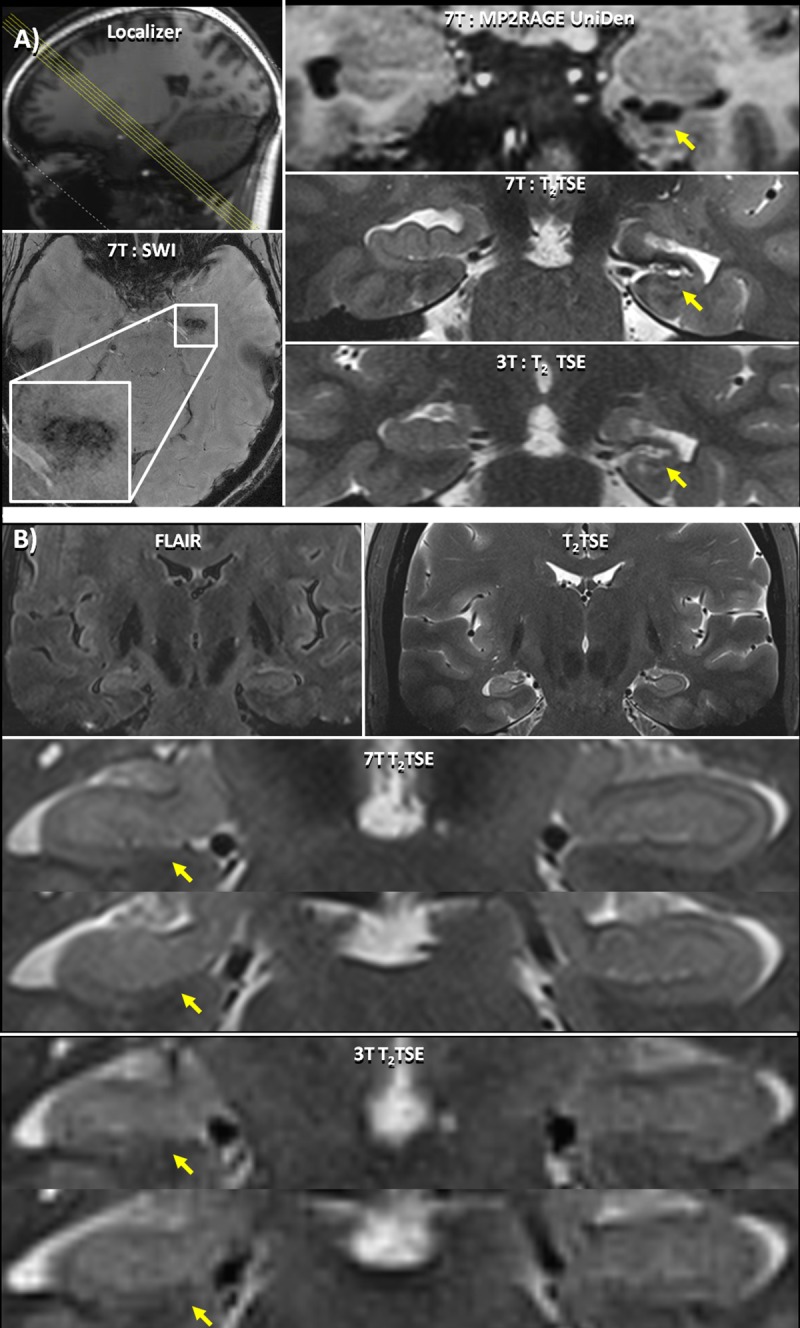
Hippocampal Asymmetry. (A) Patient 19 –clockwise from top left: A low resolution localizer indicating the coronal-oblique slice thorough the hippocampus shown; 7T: MP2RAGE UniDen reconstruction visualizing the cavity; 7T:T_2_ TSE image showing a coronal oblique slice through the hippocampus and a visualization of the parenchymal cavernoma; 3T: T_2_ TSE scan, acquired previously, showing the location of the lesion. On the 3T image, the lesion was less conspicuous and therefore went undiagnosed despite being identified in a retrospective examination of the image; SWI axial slice where the cavernoma can be clearly identified. (B) Patient 24—from top left: A 7T FLAIR image showing relatively equivalent signal intensity in both hippocampi; 7T:T_2_ TSE image showing full coronal-oblique slice and right hippocampal sclerosis, and 3T T_2_ images showing the hippocampus.7T:T_2_ TSE slice series showing a coronal oblique slice through the hippocampus showing right hippocampal sclerosis with decreased digitation and lamination without accompanying signal change in the hippocampus on the FLAIR image. The 3T T_2_ images for this subject do not show this architectural change in the hippocampus.

**Fig 2:**
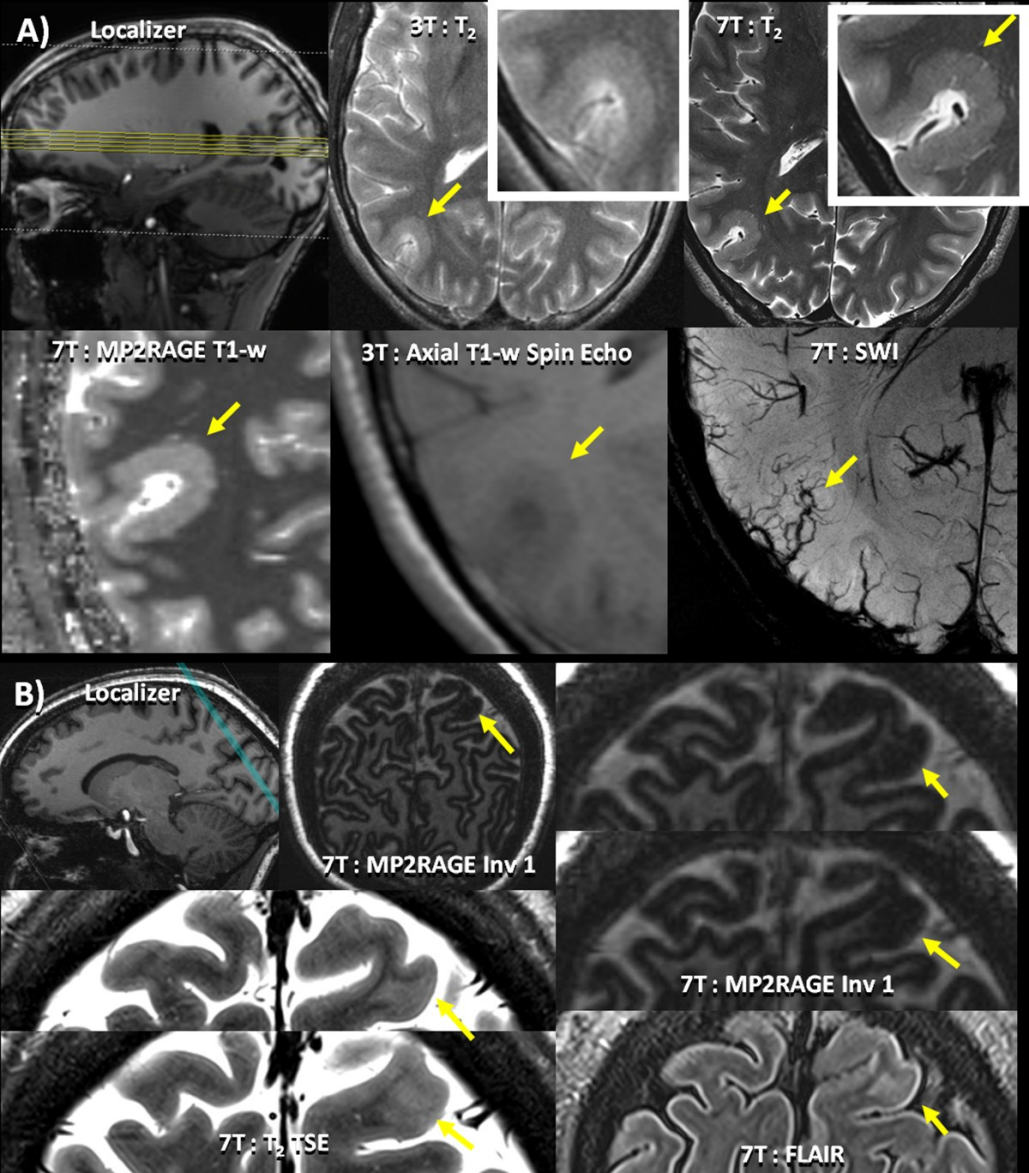
Cortical Abnormalities. (A) Patient 7 –clockwise from top left: Localizer image showing the location of the axial slices; 3T T_2_ axial image of the lesion illustrating subtle changes in cortical thickness detected only after the lesion was identified at 7T; 7T T_2_ TSE slice visualizing the polymicrogyria marked by a yellow arrow highlighting the texture of the polymicrogyria; 7T: MP2RAGE with T1 weighted reconstruction highlighting the abnormal thickening of the cortex due to the polymicrogyria; 3T T1-w spin-echo of the same region; 7T SWI axial slice showing abnormal vasculature due to the polymicrogyria (B) Patient 36 –clockwise from top left: Localizer image showing the location of the axial slices; MP2RAGE full coronal-oblique slice showing cortical dysplasia (yellow arrow) in the left parietal lobe; enlarged slices of 7T MP2RAGE image showing cortical dysplasia marked by a yellow arrow in the left parietal lobe; 7T FLAIR slice showing the location of the cortical dysplasia (yellow arrow) enlarged slices of 7T T_2_ TSE image showing cortical dysplasia (yellow arrow) in the left parietal lobe.

**Fig 3:**
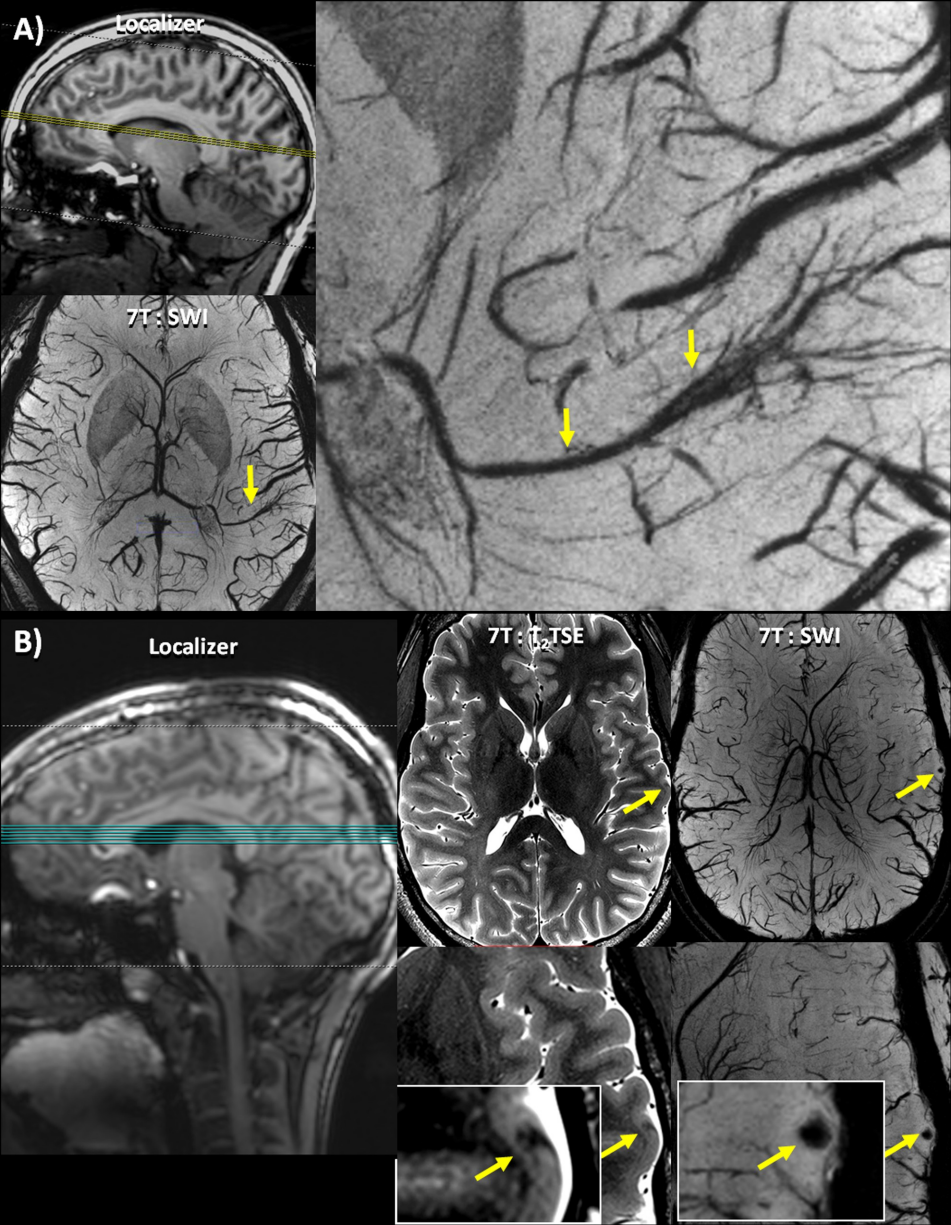
Lesions identified on SWI. (A) Patient 17 –clockwise from top left: Localizer image showing the location of the axial slices; an enlarged view of a DVA associated with the sSOZ identified on the SWI; full axial slice of 7T SWI minimum intensity projection showing a DVA.(B) Patient 10 –left to right: Localizer image showing the location of the axial slices; T_2_ TSE slice (full slice above, enlarged image below) showing a cortical thickness defect indicated by a yellow arrow, initially identified on SWI; SWI slice (full slice above, enlarged image below) showing a punctate focus of susceptibility indicated by a yellow arrow co-localized with a cortical thickness defect.

No subjects were excluded due to image quality. Final MRI observations are shown in Table 3, along with the patient age, sex and sSOZ. Examples of abnormalities detected at 7T, which were not detected at clinical field strengths, are shown in Figs 1, 2 and 3.

**Table 3:** Summary of epilepsy patient results.

ID	Sex [M/F] Age [Years]	Suspected Seizure Onset Zone	Clinical Scan [1.5/ 3.0]T	7T Report (Blinded)	Additional 7T Report (Unblinded)	Relation to sSOZ
1	M 28	Left frontotemporal	1.5T	DVA R frontal horn Partial duplication of R lateral transverse sinus		None
2	M 19	Non-lateralizable frontal	1.5T	Tortuous distal cervical carotid arteries (R>L) Pineal cyst Prominent PVS in: Parietal white matter (R>L) Superior frontal gyrus (L>R)		Uncertain
3	M 33	Bilateral frontotemporal	3.0T	Empty sella Symmetrical prominent PVS	Bilateral small hippocampi Bright hippocampi	Possible
4	M 56	Left hemispheric Anterior	1.5T	R cerebellar cavernoma Prominent arachnoid granulations, around SSS Cord plexus cysts in ventricles (L>R) Brain volume loss AA		None
5	M 20	Bilateral: Right >> Left frontal	3.0T	Asymmetry of lateral ventricles (L>R) Asymmetry of ventricle atria (R>L) Prominent PVS (R>L)		Uncertain
6	F 33	Right temporal	3.0T	Hippocampal asymmetry (R>L) Ventricle asymmetry (R>L) Temporal horn asymmetry (L>R) Empty sella Frontal hyperostosis frontalis, age atypical Meckel’s + oculomotor ectasia Prominent symmetrical PVS–parietal		Uncertain
7	M 25	Right hemispheric frontal and parietal onset	3.0T	R temporal occipital cortical polymicrogyria Pial hypervascular area on SWI correlating with cortical abnormality		Definite
8	M 40	Left temporal (probable)	3.0T	DVA L frontal gyrus Ventricular asymmetry (R>L) Partially empty sella Prominent arachnoid granulations, SSS Brain volume loss AA Symmetrical prominent PVS		None
9	M 25	Left temporal onsets Bilateral temporal interictal epileptiform potentials	1.5T	Normal	R hippocampal architecture disruption	Uncertain
10	M 19	Right frontotemporal	3.0T	Prominent occipital PVS (L>R) L punctate cortical SWI focus subcentral gyrus region	L cortical thickness defect	Uncertain
11	F 56	Bilateral multifocal	3.0T	Asymmetric ventricular atrium (R>L) Prominent PVS (L>R)		None
12	M 39	Left frontotemporal	1.5T	DVA R middle frontal gyrus Brain volume loss age atypical Prominent occipital PVS (R>L)		Uncertain
13	M 37	Left temporal	1.5T	Signal increase in left hippocampus	Hippocampal asymmetry	Possible
14	M 43	Bilateral temporal	1.5T	R hippocampal architecture disruption L thalamus DVA Ventricle asymmetry (L>R)		Possible
15	M 29	Non-lateralizable frontal	3.0T	Conspicuous arachnoid granulations		None
16	F 28	Left frontal	1.5T	Cerebellar volume loss Symmetrical prominent PVS		None
17	M 29	Left posterior temporal/parietal	1.5T	R cerebellar corpus medullare DVA Ventricle asymmetry (R>L) Prominent arachnoid granulations at vertex	L parietal DVA	Possible
18	F 45	Left temporal	1.5T	SWI focus in medial R temporal lobe Ventricle asymmetry (L>R) Prominent symmetrical PVS, low basal ganglia		Uncertain
19	F 27	Left temporal	1.5T	L hippocampal architecture disruption Cavernoma in L mesial temporal lobe	Hippocampal asymmetry (R>L)	Likely
20	F 30 51	Left frontal	1.5T	Ventricle asymmetry (R>L)		None
21	M 51	Right temporal	1.5T	SWI focus subcortical L mesial temporal Symmetrical PVS basal ganglia Prominent arachnoid granulations SSS	Mild hippocampal asymmetry, increased signal and volume (R)	Possible
22	M 23	Non-lateralizable frontal/parietal	1.5T	R atrial periventricular leukomalacia	Cortical irregularity; pars marginalis, subtle SWI (R)	None
23	M 45	Left anterior temporal	1.5T	L hippocampal architecture disruption, cyst Symmetrical PVS basal ganglia		Likely
24	F 32	Right anterior/mesial temporal	3.0T	R hippocampal hyperintensity R hippocampal decrease in digitation Partial duplication of SSS Pineal cyst Prominent PVS R basal ganglia	Hippocampal sclerosis (R CA1-4 decreased thickness) Decreased hippocampal lamination (R)	Definite
25	F 33	Bilateral mesial temporal	3.0T	Partially empty sella Prominent PVS L sub insula Prominent PVS R fronto-parietal		None
26	F 48	Bilateral temporal	3.0T	Patchy regions of white matter signal increase Prominent PVS L parietal, occipital, and frontal		None
27	M 78	Left temporal	1.5T	Marked increase in ventricle > sulci Hydrocephalus	Bilateral hippocampal atrophy	None
28	M 65	Left mesial frontal	1.5T	Decreased volume L hippocampal body R frontal DVA Ependymitis granularis Symmetrical PVS	Decreased volume L hippocampus (subiculum, CA1, CA2)	Uncertain
29	F 40	Left frontal	1.5T	R choroid tissue hippocampal architecture disruption Superior cavum ventricle asymmetry Prominent arachnoid granulations SSS, R transverse sagittal Internal auditory canal ectasia Prominent PVS L parietal	Possible dysplasia (L posterior inferior frontal) Asymmetrical medial occipital sulcation around posterior hippocampus	Possible
30	F 28	Right frontal/temporal/parietal	1.5T	Decreased volume R hippocampus; hyperintense FLAIR signal L frontal DVA Prominent arachnoid granulations L transverse sagittal Focal periatrial T_2_ signal increase L > R Prominent PVS L subinsular		Possible
31	F 29	Left unknown	1.5T	Possible ependymal granulations	Hippocampal asymmetry (L slightly decreased)	Uncertain
32	F 22	Right multiple onset mesial/lateral and generalized	1.5T	Decreased digitation L anterior hippocampus R superior cervical tortuous carotid arteries Symmetrical PVS	R hippocampus is slightly taller Increased undulation of inferior margin	Uncertain
33	F 34	Left posterior parieto-occipital	1.5T	Hippocampal asymmetry R>L; slight R signal hyperintensity Cortical defect L occipital lobe Prominent arachnoid granulations L transverse sinus	Increase of subcortical signal inferior calcarine (L)	Likely
34	F 65	Left temporal	3.0T	Hippocampal asymmetry R> L; L hippocampal head architecture disruption Decreased cortical lamina anterior L hippocampus Prominent arachnoid granulations parasagittal vertex Inferior tentorium meningioma White matter ischemic disease Symmetrical PVS	Hippocampal hyperintensity (L) Diminished volume parahippocampal white matter (L)	Definite
35	F 23	Left fronto-temporal	1.5T	Terminal myelination Parietal/occipital ventricle asymmetry Prominent PVS R basal ganglia		None
36	F 35	Left parietal	1.5T	Mild R hippocampal signal hyperintensity Left parietal cortical dysplasia Prominent PVS R superior periatrial		Definite
37	M 24	Right anterior temporal	3.0T	R medial temporal cavernoma Symmetrical PVS		Definite

Suspected seizure onset zones, previous MRI field strength, 7T report, and the relationship between the 7T report and the suspected seizure onset zone. Abbreviations -> AA: age atypical; DVA: developmental venous anomaly; F: female; M: male; L: left; R: right; PVS: perivascular spaces; sSOZ: Suspected Seizure Onset Zone; SSS: superior sagittal sinus; SWI: susceptibility weighted imaging.

In total, 25 potentially epileptogenic abnormalities were identified at 7T that were undetectable or otherwise overlooked at lower field strengths. 7T findings were *definitely* related to the patients' epilepsy in five patients (Table 3: #7, #24, #34, #36, #37; Figs 1B and 2), *likely* related in three patients (Table 3: #19, #23, #33; Fig 1A), *possibly* related in seven patients (Table 3: #3, #13, #14, #17, #21, #29, #30; Fig 3A), and *uncertain* in ten patients (Table 3: #2, #5, #6, #9, #10, #12, #18, #28, #31, #32; Fig 3B).

**Fig 4:**
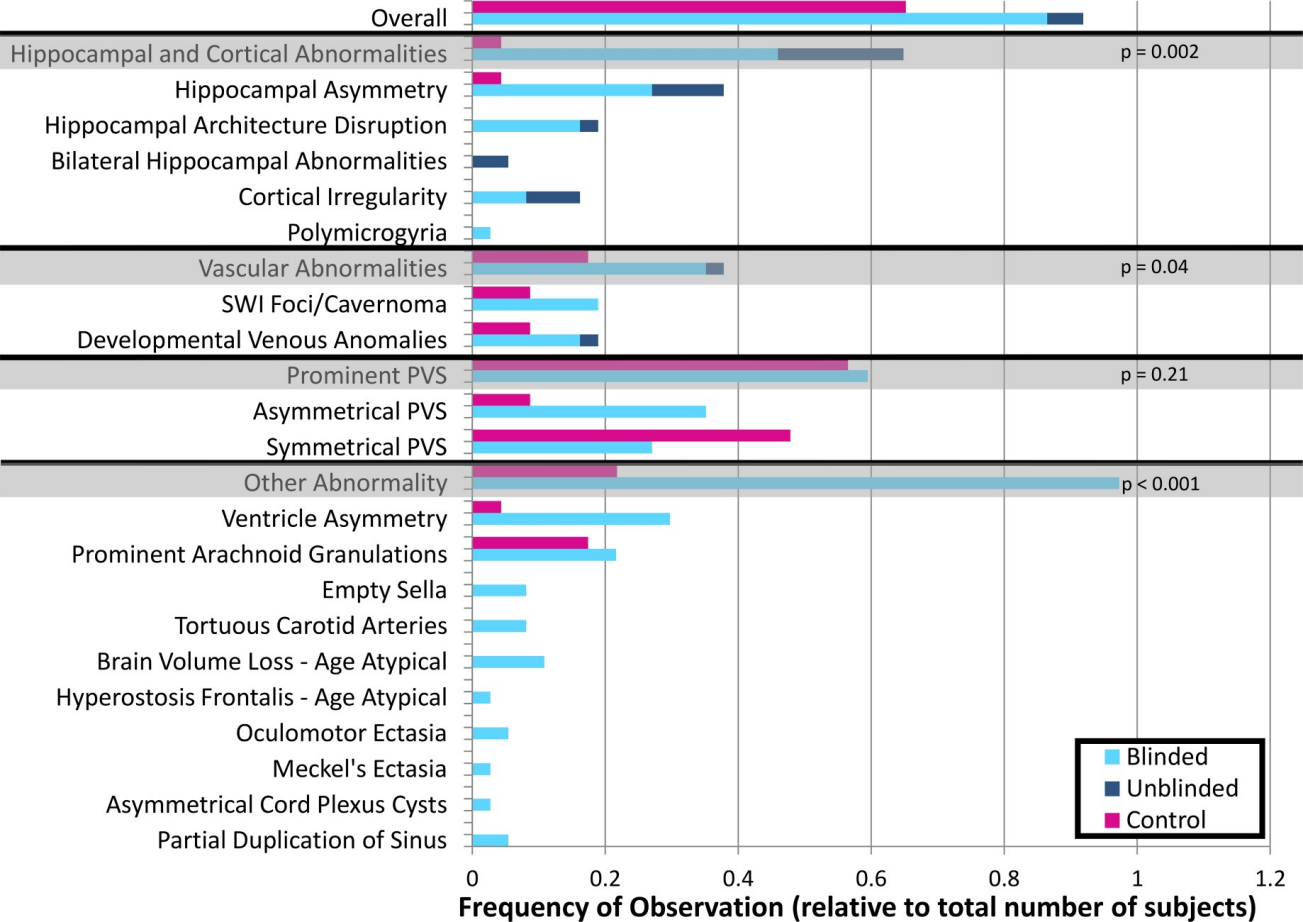
Lesion frequency. Graph showing numbers of reported findings in both controls (pink) and patients with epilepsy when blinded (light blue), and unblinded (dark blue). Grey shaded rows show total numbers for a particular category of findings. Abbreviations: PVS-perivascular spaces; SWI–susceptibility weighted imaging.

A range of structural and vascular abnormalities or findings were reported in all patients and 17 (out of 21) healthy controls. In 36 patients the radiologist report indicated a finding when the images were read blinded to the patients’ previous clinical history (blinded). In 16 patients, additional radiological findings were identified when the status and previous clinical history were revealed to the radiologist (unblinded). These results are summarized in Table 3 by patient and organized by finding type in a bar chart (Fig 4). The total number of abnormalities, classified by type, in patients (blinded/unblinded) and healthy controls were: hippocampal asymmetry (blinded/unblinded patients = 9/4; controls = 1), hippocampal architecture disruptions (blinded/unblinded patients = 6/1), polymicrogyria (blinded patients = 1), bilaterally small hippocampi (unblinded patients = 2), other cortical irregularity (blinded/unblinded patients = 3/3), SWI foci/cavernomas (blinded patients = 7; controls = 2), and developmental venous anomalies (DVAs) (blinded/unblinded patients = 6/1; controls = 2). In some patients more than one finding was seen. These findings were either not visible, ambiguous, or not described in the previous lower-field clinical exams. Other potentially incidental findings in patients included prominent perivascular spaces, partially empty sella, Meckel's ectasia, oculomotor ectasia, and ventricular asymmetry.

### Comparison with clinical exam

In the eight patients with *definite* or *likely* lesions identified on the 7T MRI, retrospective evaluation of the clinical images revealed evidence of all or part of these abnormalities in three patients. In Patient 7 (Fig 2A) the polymicrogyria appears as a thickening of the cortex that was missed in the initial clinical read. However, details of the polymicrogyria were clearly visible in the 7T images. In Patient 19 (Fig 1A) and Patient 37 the cavernoma was subtly present in the clinical scan but was misidentified on the clinical evaluation and the additional hippocampal asymmetry was not appreciated. In an additional four patients (#23, #24, #33, #34) with *definite* or *likely* lesions related to hippocampal architecture disruptions or asymmetry, high resolution visualization of hippocampal subfields and lamination, possible at 7T but not 3T, was integral to the assessment. In two patients with *likely* and *definite* lesions (#33, and #36) the cortical abnormalities were not visible in their clinical scans. However, the scans were performed at 1.5T and it is possible that they would be visible at higher clinical field strength. Thus, in cases where the evidence of abnormalities is present in retrospective analysis of the 3T images, the increased resolution and contrast at 7T has made accurate identification easier.

### Comparison with healthy controls

In the healthy controls, subtle abnormalities were also detected but were less frequent when compared to the blinded assessment of epilepsy patients (Fig 4). The difference in the abnormality counts on the blinded review was found to be significant in three categories: a) hippocampal and cortical abnormalities (p = 0.002, sensitivity = 0.51; specificity = 0.95), b) vascular abnormalities (p = 0.04, sensitivity = 0.40, specificity = 0.81), and c) all other aggregated abnormalities identified on T_2_ TSE, MPRAGE, MP2RAGE, and FLAIR (p<0.001, sensitivity = 0.70, specificity = 0.70). No significant difference in reported prominent perivascular spaces was found between the two groups (p = 0.21, sensitivity = 0.68, specificity = 38). The frequency with which these findings occurred in both healthy controls and epilepsy patients is shown in Fig 4.

### Hippocampal and cortical abnormalities

#### Hippocampal asymmetry

The 7T images for 13 epilepsy patients (Table 3: #6, #13, #19, #21, #24, #28, #29, #30, #31, #32, #33, #34, #36) showed hippocampal asymmetry (reduced volume visually assessed in a part of the hippocampus in one hemisphere when compared to the other hemisphere), frequently without definite T_2_ signal change. An example of asymmetry between the right and left hippocampal structures can be seen in the 7T T_2_ TSE and MP2RAGE of patient 19 (Fig 1A). In patient 24 (Fig 1B), the T_2_ TSE shows a right hippocampal sclerosis, with increased signal and decreased digitation and lamination. A mild (not clinically significant) hippocampal asymmetry was also noted on the MPRAGE images of one healthy control.

Eight of the patients with hippocampal asymmetry had epilepsy of suspected temporal origin (Table 3: #6, #13, #19, #21, #24, #30, #32, #34). The hippocampal abnormality was rated the *definite* or *likely* cause of epilepsy in patients 19, 24 and 34. The abnormality was on the same side and in the same location with the sSOZ (rated: *possible*) in three more patients (Table 3: #13, #21, and #30). However, the abnormality was not thought to be the definite cause in these cases. In patients 6 and 32, the hippocampal asymmetry was of *uncertain* significance (rated: *uncertain*). In patient 6, the sSOZ was in the temporal lobe with the larger hippocampal volume without signal change on either side. Patient 32 had seizures with multiple onsets, and while the asymmetry was not solely responsible for seizure onset, the hippocampal involvement was considered to be part of the patient’s overall disease profile.

#### Other hippocampal abnormalities

Seven patients (Table 3: #9, #14, #19, #23, #24, #29, #34) had disruptions in the hippocampal architecture and two patients (Table 3: #3, #27) had bilaterally small hippocampi. In three patients (Table 3: #9, #14, #23) hippocampal architecture disruption was noted without a marked qualitative asymmetry in the hippocampal volume. No hippocampal architecture disruption or brightening was noted in the healthy controls.

Patient 3 had bilaterally small, bright, hippocampi when visualized on the T_2_ TSE and MPRAGE images. In patient 27, the hippocampal atrophy was not suspected to be epileptogenic, but was part of generalized brain volume loss that was atypical for the patient’s age.

All but one of the patients (Table 3: #29) with other hippocampal abnormalities had epilepsy of temporal origin. Of the patients with hippocampal architecture disruption or bilaterally small hippocampi the finding detected at 7T was rated as the *definite* or *likely* cause of epilepsy in three patients (Table 3: #23, #24, #34); was correlated with the sSOZ and rated the *possible* cause of epilepsy in two patients (Table 3: #3, #14); and was rated *uncertain* as the cause in patient 9, for whom the sSOZ was in the contralateral temporal lobe.

#### Other cortical abnormalities

In patient 7, a polymicrogyria was first detected in the right temporal occipital cortex on the 7T axial T_2_ TSE and coronal-oblique MP2RAGE images. The cortex at the polymicrogyria was thickened to between 5.05 and 6.7 mm; the surface at the white matter / gray matter boundary appeared blobby and thin microgyria were apparent at both interfaces. Increased abnormal venous vasculature co-localized to this cortical defect on the 7T SWIs (Fig 2A). The polymicrogyria was rated by the epileptologists as the *definite* cause of epilepsy in this patient. Pathology confirmed the presence of a focal cortical dysplasia with an area of molecular layer in-folding with projection into the subjacent cortex consistent with the features of a polymicrogyria. Polymicrogyria was not identified in any of the healthy controls.

A cortical defect was detected in the MP2RAGE images in the left occipital lobe in patient 33. In patient 36, a left parietal cortical dysplasia was detected in the MP2RAGE image (Fig 2B).

In patient 10, a punctate focus of susceptibility was initially detected on the SWI, and associated with a subtle defect in the cortical thickness in the T_2_ TSE, not concordant with the patient's sSOZ (rated: *uncertain*). No similar defects were noted in the healthy controls.

#### Vascular abnormalities

Cavernomas were identified in three patients (Table 3: #4, #19, and #37). In patients 19 and 37, the cavernoma was associated with the sSOZ (rated: *likely* and *definite*); while in patient 4, the cavernoma, located in the cerebellum, was not associated with the sSOZ (rated: *none*). SWI foci, which could be potential cavernomas, were also noted in two healthy controls (Table 4: #8, #17). Of note there were 4 more patients with SWI lesions not thought to be cavernomas or developmental venous abnormalities (Table 3: #7, #10, #18, #21).

**Table 4:** Summary of MRI findings in controls.

Healthy Control	Gender	Age at scan	7T Report
1	Female	28	Symmetrical prominent PVS
2	Male	40	Symmetrical prominent PVS + Hippocampal asymmetry (R>L)
3	Male	20	Symmetrical prominent PVS
4	Male	25	Asymmetrical prominent PVS
5	Male	33	Symmetrical prominent PVS + Ventricular asymmetry (L>R)
6	Male	39	Symmetrical prominent PVS + Oculomotor ectasia (L)
7	Female	33	Symmetrical prominent PVS + DVA–R temporal
8	Male	37	SWI focus/cavernoma
9	Male	19	Normal
10	Male	28	Symmetrical prominent PVS + DVA–R precentral gyrus
11	Male	20	Symmetrical prominent PVS
12	Male	25	Meckel’s ectasia
13	Male	29	Mild ventricle asymmetry (R>L)
14	Male	43	Moderately prominent arachnoid granulations
15	Female	56	Oculomotor ectasia
16	Female	30	Symmetrical prominent PVS
17	Female	27	Symmetrical prominent PVS + Tiny SWI focus/cavernoma
18	Male	56	Normal
19	Female	32	Normal
20	Male	44	Normal
21	Male	48	Symmetrical prominent PVS

Abbreviations: DVA-> developmental venous anomaly; L—> left; R -> right; PVS-> perivascular spaces.

DVAs were detected on the SWI images in seven patients (Table 3: #1, #8, #12, #14, #17, #28, #30) and two healthy controls (Table 4: #7, #10). In patients 17 and 30, a DVA localized to the electrographic abnormality (rated: *possible*). In patient 12, the DVA was rated with *uncertain* relation to the sSOZ, and in the remaining four patients (Table 3: #1, #8, #14, #28), the DVA was rated to have *no* relation to the sSOZ.

#### Perivascular spaces

Prominent perivascular spaces were visible on most of the axial and coronal-oblique T_2_ TSE images within both patients and healthy controls (patients = 22, healthy controls = 13), making this the most frequent finding. Interestingly, the perivascular spaces were qualitatively assessed to be more asymmetric in the epilepsy group [39].

#### Other abnormalities

Although not known to be reflective of disease pathology, a number of other findings were reported more frequently in epilepsy patients than in healthy controls. Prominent arachnoid granulations were found in both epilepsy patients and healthy controls. These granulations may have been visible but were not reported in the clinical field strength exams. In a previous study, performed at 1.5T, arachnoid granulations were observed to be present in 10% of subjects scanned [40].In our population, the incidence is approximately double, due to increased conspicuity in the T2 TSE.

Small foci of susceptibility, likely not cavernomas, were identified on SWI in three epilepsy patients (Table 3: #10, #18, #21), with *uncertain* relation to the sSOZ. In patient 10, a cortical focus of susceptibility was found contralateral to the sSOZ. A tiny focus of susceptibility artifact was found in the right mesial hippocampal structures of patient 18 which was contralateral to the sSOZ. A subcortical mesial temporal focus of susceptibility was noted in patient 21 who also had hippocampal asymmetry.

Of the remaining abnormalities noted, significant ventricular asymmetry was identified in ten epilepsy patients, but was observed in only one control.

#### Surgery

At the time of writing, ten epilepsy patients have progressed to therapeutic surgery, one subject had SEEG placement but will not proceed to therapeutic surgery based on the results and an additional three subjects have been recommended or scheduled for surgery. Of the ten completed therapeutic surgeries, five resulted in seizure-freedom (Class I), three resulted in close to seizure freedom (Class II), and the remaining two resulted in worthwhile improvement (Class III). Five patients had findings with a *definite* relationship to the sSOZ and two with a *possible* relationship to the sSOZ. Details of the use of the 7T report in surgery, type of surgery, and surgical result are available in Table 5. The subset of Class I outcomes (5/10) had 7T findings which were predominantly thought to be the *definite* cause of the epilepsy (4/5). For example for patient 36, the 7T data showed an extremely subtle left parietal cortical dysplasia. This data in conjunction with a retrospective evaluation of the clinical MRI helped guide the placement of her bilateral SEEG electrodes. The sSOZ localized to the lesion and subsequent pathology confirmed a cortical dysplasia. The patient had been having several seizures daily and is now seizure free.

**Table 5:** Summary of epilepsy patients progressing to surgery and utility of 7T information for surgical intervention.

ID	sSOZ	Use of 7T Report	Relation to sSOZ	Type of Surgery	Pathology	Surgical Outcome (Engel Scale)
5	Bilateral: Right >> Left Frontal	Suggested investigations into multi-focal nature disease	Uncertain	Intracranial electrodes then RNS	N/A	3
7	Right hemispheric Frontal and parietal onset	Permitted retrospective identification on previous scan permitting progression to surgery	Definite	Grid + Resection of polymicrogyria	Focal Cortical Dysplasia excision with focal features of polymicrogyria and gliosis	2
11	Bilateral Multifocal	Anatomical planning and electrode placement	None	SEEG then VNS	N/A	2
13	Left Temporal	Anatomical planning and electrode placement	Possible	SEEG then RNS	N/A	3
17	Left Posterior temporal/parietal	Recommended for surgical intervention	Possible	Recommended for surgery	N/A	N/A
19	Left Temporal	Changed surgical prognosis	Definite	Recommended for surgery	N/A	N/A
21	Right Temporal	Invalidated previous, artifactual, findings.	Possible	Grid + Resection of right lateral temporal lobe	Scattered neurons in the subcortical white matter consistent with heterotrophic neurons of uncertain epileptogenic significance	1
24	Right Anterior/mesial temporal	Assisted in the placement of electrode placement.	Definite	SEEG and laser ablation	N/A	1
25	Bilateral Mesial temporal	Not used	None	b/l SEEG b/l hippocampus/amygdala RNS	N/A	2
28	Left Mesial-Frontal	Used to identify targets for placement of SEEG	Uncertain	SEEG, no theraputic stage recommended	N/A	N/A
34	Left Temporal	Confirmed EEG findings and aided in surgical planning	Definite	Laser ablation	N/A	1
35	Left Fronto-temporal	Not used	None	b/ SEEG + Scheduled RNS	N/A	N/A
36	Left Parietal	Confirmed EEG findings and changed surgical prognosis	Definite	Left parietal focal resection	Brain lesion: ganglioglioma with atypical features	1
37	Right Anterior Temporal	Aided in surgical planning	Definite	Resection of temporal tip	Pathology Report Not Available	1

Abbreviations -> b/l: bilateral; EEG: electroencephalography; N/A: not applicable; RNS: responsive neurostimulation; SEEG: stereo electroencephalography; sSOZ: Suspected Seizure Onset Zone; VNS: vagus nerve stimulation.

## Discussion

The high resolution and enhanced contrast afforded by imaging at 7T enabled the detection of potentially epileptogenic abnormalities (*definite* = 5; *likely* = 3; *possible* = 7; *uncertain* = 10) that were undetectable or otherwise overlooked at lower field strengths. The high resolution T_2_ TSE images were most frequently used for initial detection of subtle structural abnormalities at 7T. The FLAIR images were used primarily to note signal hyperintensities associated with hippocampal sclerosis. The first inversion time reconstruction of the MP2RAGE facilitated the detection of cortical lesions. The remaining MP2RAGE reconstructions and the MPRAGE images were valuable to confirm and categorize findings. Isotropic images can be re-sliced in any plane to optimize evaluation of structural asymmetries. 7T SWI allowed the effective visualization of irregular vasculature associated with cortical abnormalities, such as polymicrogyria.

### Patient inclusion

Three patients had completely normal EEGs at the time of enrollment into the study (patients 15, 28 and 29). Patient 15 had nocturnal hyperkinetic seizures from sleep with clinically focal features and recent hospitalization with a bitten tongue and elevated CPK. A seizure was not captured on EEG and interictal EEGs were normal. Patient 28 had normal EEG’s until after enrollment when multiple electrographic seizures were captured, and patient 29 had frequent focal aware seizures (FAS) of right facial twitching which were surface negative.

### T_2_ TSE: Hippocampal abnormalities

In the epilepsy patient group, 23 hippocampal abnormalities were found in 18 patients. The abnormality was concordant with clinical and EEG data in 12 of these 18 patients with hippocampal abnormalities.

In two of the remaining six patients in which the abnormality did not correlate with clinical and EEG data, the sSOZ was considered to be unknown (Table 3: #31, #32). In two additional patients, the 7T MRI lesions were found contralateral to the sSOZ. It is possible that this apparent discordance may be an indicator of complex seizure onset for these patients. For example, one patient (Table 3: #13) had an abnormal appearing right hippocampus on MRI but seizures were of left temporal onset (Table 2: #13). However, during an intracarotid amobarbital (Wada) test, the right-sided memory score was very poor whereas the left-sided memory score was nearly perfect. This pattern is the opposite of what is expected in someone with left temporal epilepsy, suggesting right temporal pathology. Another patient, whose sSOZ was contralateral to the hippocampal abnormality (Table 3: #9), was suspected to have frontal lobe epilepsy instead. This patient was refractory with frequent seizures. It is possible that this condition led to secondary hippocampal damage.

### T_2_ TSE: Perivascular spaces

The most frequent observation was the prominence of perivascular spaces on T_2_ TSE images. This finding was common and highly non-specific, noted in both epilepsy patients and controls (specificity = 0.38, sensitivity = 0.68). This is likely because of the improved contrast and resolution at 7T, lowering the threshold of detectability for small features. Although the appearance of perivascular spaces in the high resolution images was not significantly different between groups, the distribution was qualitatively observed to be asymmetrically clustered to one hemisphere in epilepsy patients more frequently than in healthy controls. The qualitative results reported here agree with the results of a quantitative analysis showing that asymmetry of perivascular spaces is related to the sSOZ in epilepsy[39].

### Susceptibility weighted imaging

SWI was a particularly informative component of the protocol. The SWI sequence was highly sensitive for the detection of focal susceptibility changes caused by or associated with cortical defects, cavernomas and DVAs. Although DVAs are often considered benign, they have previously been reported in association with cortical dysplasias and epilepsy [19, 30, 41–43]. In our experiment, abnormal susceptibility signal was associated with the sSOZ in five epilepsy patients. In some cases, an abnormality first visualized on SWI, led to focused review of the structural scans in the vicinity and resulted in improved detection of potential epileptogenic abnormalities.

In one patient with refractory temporal lobe epilepsy (Table 3: #19), a left mesial cavernoma detected in the SWI corresponded with a diminished and distorted left hippocampus (Fig 1A). These findings co-localized with left temporal seizure onset on EEG. This same lesion was read as a choroidal fissure cyst on the patient’s prior MRI at 3T. In this case, finding the cavernoma facilitated management for this patient. Our overall SWI findings support previous research indicating that 35%-70% of symptomatic cavernomas are associated with seizures [44, 45]. Specifically, previous studies have shown that resection of cavernomas often results in seizure freedom, with the greatest success being in mesial temporal cavernomas [46, 47].

In a second patient (Table 3: #7), an SWI finding co-localized to a polymicrogyria. The seizures did not lateralize on EEG, but the 7T finding was confirmed on his 3T scan, leading to more definitive lateralization of seizure onset. In this case, the treatment plan was changed from a bilateral intracranial strip study to a unilateral two-stage intracranial study that resulted in successful resection of the lesion and obviated the need for a second surgery. Although the polymicrogyria was retrospectively identifiable on 3T (Fig 2A), the lesion was not detected prospectively. This may be because resolution at 3T was insufficient to properly characterize and delineate the cortical border, resulting in reduced conspicuity of the abnormality. In the remaining three patients, the SWI findings (2 DVA’s and 1 cavernoma; Table 3: #17 #30, #37 respectively) localized to the EEG findings.

### Comparison of epilepsy versus healthy controls

The higher resolution and improved contrast at 7T led to increased conspicuity of brain structures in both epilepsy patients and healthy controls. Clinically significant results such as hippocampal and cortical abnormalities (p = 0.002) as well as vascular abnormalities of all types (p = 0.04) were more common in the blinded reads epilepsy group (Fig 4). Hippocampal and cortical abnormalities were present in about half the epilepsy patients and were the most specific finding, with a single hippocampal asymmetry detected in healthy controls (specificity = 0.95, sensitivity = 0.51). Separately, the detection of DVAs in healthy controls reduced the specificity of these findings (specificity = 0.81, sensitivity = 0.40). Other abnormalities (exclusive of perivascular spaces) that were not related to epileptogenic foci were, nonetheless, significantly more frequent in epilepsy patients (p<0.001). Therefore, abnormalities seemingly unrelated to seizures, although less specific than hippocampal and cortical abnormalities or vascular abnormalities, are the most sensitive neuroradiological findings in this population (specificity = 0.71, sensitivity = 0.70). Future quantitative analysis of these structural findings may result in the identification of biomarkers for the pathogenesis of the disease. The use of healthy controls helped to differentiate epileptogenic findings from non-pathological findings that became detectable due to the resolution and contrast advantage afforded at 7T.

### Surgical outcomes

In nine of ten patients that progressed to therapeutic surgery, the 7T findings were helpful in guiding a retrospective analysis of diagnostic test results, validating sSOZs, and assisting surgical planning when evaluated in addition to clinical standard of care imaging (Table 5: “Use of 7T Report”). Four of these surgeries resulted in seizure freedom (Table 5: #21, #34, #36) and one resulted in almost seizure-freedom (two seizures in two years post surgery; Table 5: #7). Patient #36 is a good example where the seizure semiology pointed to a left brain pathology consisting of an abnormal feeling followed by right body movements. Her scalp EEG showed bilateral left > right, spikes but her 7T MRI showed a left parietal cortical dysplasia. Subsequent intracranial SEEG tailored to the lesion demonstrated 12 seizures with left parietal onset. The patient underwent resection of the lesion previously illustrated by the MRI and the pathology showed a ganglioglioma with atypical features.

In the remaining cases that progressed to therapeutic surgery, intracranial grids and strips or SEEG leads were placed first, followed by additional surgical intervention (responsive neurostimulation, vagus nerve stimulation, or laser ablation). In these cases, the 7T MRI revealed one abnormality with a definite relationship to the sSOZ (Table 5: #24), one possible relationship to the sSOZ (Table 5: #13) and one uncertain relationship to the sSOZ (Table 5: #5). For these patients, the 7T MRI findings, when used in addition to clinical data, were helpful in more thorough and accurate placement of electrodes to validate sSOZs that were determined through clinical data.

Finally, in the single potential surgical case whose treatment plan changed after the placement of SEEG (Table 5: #28), the 7T was used to identify anatomical targets for the SEEG placement. The result of this test was the detection of a wide network of activity with multi-focal seizure onset and no definitive target for responsive neurostimulation (RNS).

Five of the surgical patients had a lesion found on 7T MRI thought to *definitely* represent their seizure onset zone (Table 5: # 7, #24, #34, #36, #37). All of these patients had excellent outcomes with three being seizure free and one having rare non-disabling seizures.

### Limitations

This study was limited by the diagnostic scan field strength at the time of enrollment. A subset of the patients scanned at 7T had received a diagnostic clinical scan at 3T, while the rest received 1.5T clinical scans. The scanning protocol used in the clinical scan was determined by the standard epilepsy protocol at the site of the diagnostic scan, and our inclusion criteria required that this scan be read as non-lesional. Retrospective re-examination of some of the diagnostic scans (ex. Patient 7) suggests that a second, higher quality 3T scan may also have detected the abnormality detected at 7T. However, in all cases, a second diagnostic scan at clinical strength had not been recommended as the existing diagnostic images had been considered sufficient by the patient’s care team. The focus of this study was to evaluate the utility of 7T as an additional noninvasive imaging test above and beyond current clinical standard of care and to compare the 7T results of epilepsy patients to those of healthy controls. The 7T detected additional abnormalities in both subsets of patients, including those scanned at 3T. Furthermore, the number of other abnormalities (exclusive of prominent perivascular spaces) that were detected was significantly greater than in healthy controls.

Additionally, the population of non-lesional patients enrolled in the study was heterogeneous, reflecting, in part, the heterogeneity of the epilepsy population. This limited the ability of the study to assess the impact of lesion detection at 7T on the efficacy of surgical intervention. However, the focus of this study was to evaluate the utility of 7T in detecting subtle abnormalities, including cortical and subcortical abnormalities. Future work in a more phenotypically homogeneous population could assess the role of these findings in influencing the efficacy of surgical intervention.

Images were collected, assessed, and added to the patient’s medical record in accordance with pre-established IRB protocols. Imaging data cannot be publicly shared because of institutional policies regarding the deposition of data in large public repositories. Data are available for researchers who meet the criteria (Mount Sinai IRB). Researchers seeking to utilize the de-identified imaging data from this manuscript should contact the Advanced Neuroimaging Research Program (ANRP) at the Icahn School of Medicine at Mount Sinai (janette.rodriguez@mountsinai.org).

## Conclusion

The improved resolution and contrast conferred by 7T MRI revealed abnormalities of epileptogenic potential in 25 out of 37 (67% of patients) patients with focal epilepsy and who had previously non-lesional clinical MRI scans at lower field strengths. 15 of these abnormalities (40% of patients) localized to the sSOZ, and the detection of seven abnormalities (19% of patients, 28% of lesions detected) contributed directly to analysis that changed subsequent surgical intervention and treatment planning. 7T MRI also revealed several subtle structural features in both patients and controls that were undetectable at lower field strengths, with significantly more abnormalities identified in epilepsy patients. Therefore, information revealed by the 7T exams has the potential to reveal biomarkers of epilepsy, provide enhanced lesion localization of focal epilepsy, increase the success of epilepsy surgery, and advance our understanding of the etiology of the disease.
